# Alcohol use during pregnancy in post-conflict northern Uganda: pregnant women’s experiences and provider perceptions

**DOI:** 10.1186/s13011-021-00418-2

**Published:** 2021-11-08

**Authors:** Apophia Agiresaasi, Nazarius Mbona Tumwesigye, Elizabeth Nabiwemba, Juliet Kiguli, Gakenia Wamuyu Maina, Goretti Nassanga

**Affiliations:** 1 College of Health Sciences Makerere University School of Public Health, Kampala, Uganda; 2 College of Humanities and Social Sciences, Makerere University School of Languages Literature and Communication, Kampala, Uganda

**Keywords:** Alcohol, Post-conflict, Women, Pregnancy, Experiences, Perceptions, Uganda

## Abstract

**Background:**

Alcohol use during pregnancy has been associated with several birth defects and developmental disabilities generally known as Fetal Alcohol Spectrum Disorders (FASD). Contextual in-depth understanding on why women drink while pregnant is scarce. For this reason, we explored pregnant women’s experiences, knowledge, attitudes as well as provider perceptions regarding prenatal alcohol consumption to inform interventions meant to address alcohol-exposed pregnancies in post-conflict settings.

**Methods:**

In the months of May and June 2019, 30 in-depth interviews were conducted with pregnant mothers who reported maternal alcohol use during pregnancy. In addition 30 Key informant interviews were carried out with health workers providing Antenatal Care services (ANC) in health facilities in Gulu, Kitgum and Pader districts in Northern Uganda. Data was recorded, transcribed and subjected to thematic content analysis.

**Results:**

Women reported diverse views regarding maternal alcohol use during pregnancy. Whereas some felt it was favourable, others had misgivings about it. There was marked variability in knowledge on dangers of drinking during pregnancy. In this study, women reported that they found themselves in alluring situations that predisposed them to drinking alcohol. These included brewing alcohol as a source of livelihood, pregnancy-induced craving for alcohol, and participation in cultural festivities that are characterised by eating and drinking alcohol. Nonetheless, women who consume alcohol during pregnancy were not held in high esteem in the Acholi communities. Various prevention interventions reportedly existed in communities to address alcohol use during pregnancy including ANC health education, public debates, radio talk shows, community health worker group and individual counselling, and local council by laws.

**Conclusions and recommendations:**

Pregnant mothers in post-conflict northern Uganda regard alcohol as a remedy to some of the social, economic and health challenges they face. Hence they continue drinking even during pregnancy because of the existing socio-cultural norms that promote it. The findings of this study demonstrate a need for sensitising communities in which pregnant women live so they can provide a supportive environment for mothers to abstain from alcohol consumption during pregnancy. Health care providers should ensure pregnant women consistently receive accurate and honest messages on the dangers of drinking during pregnancy so they can make informed decisions.

## Background

Prenatal alcohol consumption has been recognized as the cause of several congenital anomalies [1, 2]. In spite of this, alcohol consumption is generally accepted in many communities around the world [3]. In Uganda, alcohol is a fundamental part of religious and social ceremonies. A number of studies have documented higher prevalence of alcohol use among residents in Northern Uganda compared to other regions in the country [4–7]. This has partly been attributed to the traumatic stress resulting from the two-decade civil war in Northern Uganda [8]. Many people who were affected by the war including women resorted to alcohol use to manage depression while others turned to brewing and selling alcohol as a source of income to fight poverty [8].

There have also been reported increases in levels of alcohol use among women in Uganda for various reasons [4, 7]. The most recent Uganda clinical guidelines recommend alcohol history taking and health education of mothers against alcohol drinking during pregnancy. However, screening for alcohol use is not part of routine practice in health care settings in the country. The health workers depend on self-reported information [9, 10]. This is subject to under reporting due to social desirability [11].

Knowledge and attitude have been reported to influence behavior in some cases while other studies have noted that information is not synonymous with understanding [12]. A recent study in Mozambique reported that 43% of women continued to drink after they found out they were pregnant [13] while in another study in Copenhagen pregnant women reported reduction in weekly alcohol consumption during early pregnancy as compared with pre-pregnancy levels. Prevalence of alcohol use in late pregnancy in West Virginia was reported at 8.1% [14] and problem drinking was reported by 21.2% of pregnant women in Lusaka, Zambia [15].

While there is a growing body of literature on knowledge and attitudes of women on alcohol use during pregnancy, there is a dearth of qualitative investigations that articulate and contextualize the circumstances in which pregnant women find themselves that may influence their decisions to use alcohol.

The aim of this paper is to explore pregnant women’s attitudes, knowledge and experiences regarding alcohol use during pregnancy, understand perceptions of the general public in the Acholi region on alcohol use during pregnancy, and highlight available alcohol prevention approaches in the community.

## Methods

### Study area and population

This study was conducted in the Acholi sub-region in Northern Uganda, a region scarred from the two-decade protracted conflict between the Lord’s Resistance Army and the Uganda Government. For over a decade most of the populace lived in Internally Displaced Peoples (IDP) camps that affected the economic, social and moral fabric of the communities.

Acholi sub-region administratively consists of the districts of Agago, Amuru, Gulu, Kitgum, Lamwo, Nwoya, Pader and Omoro. The districts of Gulu, Kitgum, and Pader were randomly selected for this study. Acholi people constitute 4.4% (1.4million people) of Uganda’s population. Gulu district is located in the central part of Northern Uganda; Pader and Kitgum districts are located in the North East of Gulu.

Agriculture is the mainstay for locals in the three districts. Gulu district was the epi-center of much of the fighting between the Ugandan government army and the Lord’s Resistance Army. Kitgum and Pader districts also suffered many deaths and social disruption resulting from the two-decade civil war that plagued the region. Up to 32.5% of people in Northern Uganda live below the national poverty line [16]. Acholi sub-region has one of the highest domestic violence levels in the country with 52.8% of women having experienced physical violence while 59.9% experienced some form of violence including physical, emotional and sexual from their current spouse [17]. It also has a fertility rate of 5.5 children per woman. A population of 14.4% of persons aged 10 and above in Northern Uganda have never been to school [18]. Also, about 11.4% of children in the sub-region were born less than 2.5 kg, which is higher than the national average of 9.6% children born in Uganda with a low birth weight below 2.5 kg [17].

### Study design and data collection and procedures

This was an exploratory qualitative study to elucidate how pregnant women and health workers perceived alcohol use during pregnancy. It was a follow up study of a larger quantitative cross-sectional study that investigated prevalence of alcohol use among 420 women seeking ANC services in health facilities in Gulu, Kitgum and Pader districts and recorded 23.5% prevalence of alcohol use among pregnant women in this region [19].

To explore women’s perceptions, experiences and knowledge about alcohol use during pregnancy, in-depth interviews were considered the most appropriate method [20]. Pregnant women seeking ANC services who reported alcohol use during a larger quantitative survey and consented to participate in this study were interviewed. Thirty in-depth interviews (IDIs) were conducted with these women by three research assistants with skills in social and health sciences research. Prior to conducting interviews, these research assistants were trained for 2 days. They were university graduates in the field of humanities, social sciences and gender studies. They were all born and raised in the region and were proficient in the local language (Acholi). This was to ensure culturally sensitive approaches as well as make it easy to get detailed information on respondents’ experiences, perceptions and knowledge about alcohol use during pregnancy. With the help of language experts, the researchers prepared an in-depth interview guide for pregnant mothers and a Key informant interview guide for health workers in both Acoli and English. These tools were tested for conceptual equivalence and completeness in data collection with three mothers and three key informants at a health facility in Amuru district. This pilot data was analysed and formed part of the final interviews for this study.

Whereas the in-depth interviews delved deeper into the women’s experiences, knowledge and perceptions towards alcohol use during pregnancy, 30 Key informant interviews (KIIs) were conducted with health workers providing antenatal care services to obtain expert technical information and clarification on drinking patterns, alcohol prevention approaches in the community and challenges faced in fighting the vice. The KIIs also captured community views on maternal drinking during pregnancy and alcoholic beverages commonly consumed. Table 1 on Appendix 1 on page 25 shows sample questions in the data collection tools. Key informants were purposively selected based on their role in the provision of antenatal care services. Venues for both in-depth interviews and Key informant interviews were carefully selected to minimize distractions and maximise privacy for study participants. Non-participants were not allowed at these venues.

### Data management and analysis

In-depth and Key informant interviews were tape recorded, transcribed verbatim and translated to English by trained research assistants. Interview transcripts were analysed using thematic content analysis. This was chosen because of its flexibility [21]. Theme analysis allowed themes to be generated by the researcher based on literature before data analysis and previous theory and inductively from the raw data itself. Authors read through all the transcripts twice and developed a code manual. Data was then exported and systematically open-coded in ATLAS TI – 7 for content analysis. Several codes were generated. Two persons were entrusted with the coding and worked independently to ensure reliability. The coders included the first author who has a postgraduate degree in population and reproductive health and another coder who has a postgraduate degree in sociology. They both have vast experience in coding qualitative data for related studies. Disagreements between them, whenever they arose, were resolved through discussions with a third party after which some themes were collapsed into others and new codes created. When new codes were identified, previous transcripts were re-read to determine if the new codes were applicable to the texts. Thus the code manual was continually revised. These codes were then grouped into themes. A final code manual was then produced. Recurrent and emerging themes were identified and organised into meaningful categories and sub-categories [20, 22]. Some tentative themes didn’t have enough data to support them. These were broken down or accommodated in other themes [23]. Each thematic code satisfied Boyatzi’s five elements including: a conceptually meaningful label, a definition, a description of any qualifications or exclusions to the application of the theme and examples of positively and negatively coded extracts from the data. The team of investigators then met to share findings and agree on the interpretation of themes. More review and refinement was conducted to ensure coherent patterns [24]. Findings are presented using a thematic approach whereby responses from different respondents are integrated under the same theme(s). Illuminating excerpts/ quotations are used to illustrate findings.

### Ethical considerations

The study was approved by the Makerere University School of Public Health faculty Institutional Review Board and the Uganda National Council for Science and Technology (UNCST) Ref SS 4938. Written informed individual consent was sought from participants before interviews commenced. In addition, Permission was obtained from the district authorities and local leaders. Confidentiality was observed by study investigators. We assured study participants of their liberty to freely withhold information if they were uncomfortable to give it. When the recorder was used, permission of the respondents was sought before tape recording.

## Results

The study explored perspectives of women about drinking alcohol during pregnancy, their knowledge and experiences. Using the Thematic Framework Analysis (TFA), a number of themes related to the study objectives emerged from the interpretation and analysis of these results.

### Participant characteristics

Women who participated in the study were aged between 19 and 38 years. The mean age was 28. Almost all study participants were married or cohabiting save for three who had separated with partners. Eighteen were primary school dropouts, eight had attended up to secondary school, and two had completed tertiary level of education while two had never attended school. Most of the women (19) were small scale farmers, three were alcohol brewers, and two were prisoners and other two in formal employment and the rest (four) were housewives. This is presented in Table 2 in the Appendix 2 on page 27.

Majority (22) of health workers interviewed were women. Only eight were men. Most belonged to the midwifery and nursing profession, only four were medical doctors and three were clinical officers. This is presented in Table 3 on page 28 in the Appendix 3.

### Types and content of alcohol consumed

#### Commercial beverages

Respondents reported consumption of various types of alcohol. They drank bottled beer, wine, and distilled clear spirits referred to as waragi served in small plastic bags in which they are consumed. Most of these were available in bars and shops in the study areas. As expected, commercial alcohol such as beer and wine was more popular in urban areas. Two thirds of those who have attained at least secondary level of education as well as 70 % of those in the highest wealth quintile in the sample revealed that they consumed commercial alcoholic beverages. Various types of beer that were consumed included Bell lager, Nile special, Eagle extra, Club pilsner beer, Tusker, but Smirnoff came off as a more popular drink among women as it was regarded as a feminine drink. The low end users consumed cheap sachet waragi.

#### Locally made beverages

Respondents reported consumption of homemade brews such as lujutu (fermented and distilled cassava, maize or sorghum), kwete (fermented maize and/or sorghum). They also consumed Munjuti (fermented simsim), Arege (made of sorghum and yeast), Kasese (made from bananas), Uma Uma (made of yeast and sorghum flour or cassava flour) and local wine (made of yeast, tea leaves and seeds of marijuana). As anticipated, these homemade brews being indigenous are revered and consumed regularly by almost all family members. For individuals who are addicted, this alcohol is readily available as they don’t have to be purchased from the market.

### Womens’ perceptions on alcohol use during pregnancy

#### Alcohol relieves stress

Some women described alcohol use during pregnancy as favourable since they believed it helped them relieve stress resulting from other social challenges such as poverty, divorce and separation among others. They felt drinking offered them temporary recourse to their immediate concerns such as fending for their children and relatives. Given the high total fertility rate in this region, many families have many children that require feeding, clothing and school fees among others. Some women have been abandoned by their partners and have to take care of the children single-handedly.

*“It helps relieve stress. Sometimes I may have problems with my husband and I need to take alcohol to relieve myself of stress. For some women, the husbands don’t care about them. They don’t provide for their families. Some have other women. These things can cause stress,” (*IDI,Kitgum district).

#### Alcohol is medicinal

Some women held the view that pregnancy comes with ailments some of which may not be treatable by health workers. This theme emerged inductively from the data. They used alcohol to manage some of these pregnancy-related conditions such as nausea, vomiting and abdominal pains. They revealed that they were not aware of other remedies for these conditions and some who know claim do not have access to other options that can treat the ailments.

*“Actually I don’t always take alcohol when I am not pregnant but when I have nausea and feel like vomiting like you know what pregnancy does to us, I take it and it stops the vomiting. You see, I work there in the market. I can’t be vomiting everywhere or moving to the latrine to vomit all the time, otherwise I will miss or even lose some customers,” (*IDI, Pregnant Woman, Gulu district).

#### Alcohol cleanses the baby in the womb

An unexpected theme which emerged inductively from the data was the benefits of alcohol to the unborn baby. Some respondents reported that alcohol cleanses the unborn baby in the womb. They had been advised by their mothers and grandmothers to take alcohol especially waragi whenever pregnant to rid their baby of any toxic and unhealthy substances. This information has been passed on from one generation to another, so waragi is reputed as a healthy drink for pregnant mothers in these communities to the extent that they felt disregarding it would be an injustice to their unborn babies.

*“I take waragi. I just feel like taking it. They say it cleanses the baby in the womb. It cleans the baby. Our mothers, our grandmothers told us that. Every time I am pregnant I have to keep taking waragi for that reason*.” (IDI, Gulu District).

#### Alcohol use during pregnancy is inconsequential

Previous studies have associated maternal drinking during pregnancy with prior pregnancy experiences [25] and we explored this theme. Respondents believed that alcohol use, especially low level alcohol use, was without consequence. At least 60 % of multiparous women in the sample regarded drinking during pregnancy as harmless since they had been drinking during previous pregnancies and had not realized any negative impact on their offspring. These disbelieved advice from health workers about drinking during pregnancy. Even those who consumed small amounts of alcohol believed it wouldn’t affect their pregnancy in any way. They also noted that some local brews, especially those that did not contain marijuana, were less harmful than others.

*“There are women who are abusing alcohol, especially those who take too much that can affect the baby’s growth but us who take a few cups of alcohol I think we are okay. It’s those who are taking this local brew with marijuana in it that can harm the baby. I hear it is so strong that even when an adult man takes just one cup of it they begin to stammer in their speech. I have been taking alcohol before and all my children are okay,*” (IDI, Pregnant Woman, Pader District).

#### Alcohol is shameful

Yet other women considered alcohol use during pregnancy deplorable much as they admitted drinking various types of alcoholic beverages even during pregnancy. They said drinking demeans them and strips them of their dignity as women, as mothers and as caretakers.

“*Alcohol can cause shame. Sometime back I used to get embarrassed so I sat down and made a self-evaluation and that is when I said I must reduce my drinking. So I started drinking little and this time drinking from nearby or even home and one good thing is that my husband left drinking so that alone helped me a lot. As a mother you can even fail to cook and do other responsibilities. You may even forget to go for antenatal care,”* (IDI pregnant Woman Pader).

### What women know about drinking during pregnancy

#### Alcohol can harm mother and baby

Prompted by previous research findings that women know that drinking during pregnancy can harm their unborn babies [26–28], we explored women’s knowledge on alcohol use during pregnancy in this community. Findings revealed that some women possessed some knowledge on dangers of alcohol use during pregnancy although it varied from one woman to another. Some respondents had general knowledge that alcohol use during pregnancy could potentially harm the mother and unborn baby in various ways. Many said they may get accidents while drunk and end up hurting various body parts of the foetus and the mother.

*“It can be something bad. One can fall down when drunk and hurt the baby. You never know which part of the baby may get damaged, it could be the limbs and the child is born with deformed limbs. It could be the head and the child is born with a crooked head or dysfunctional brain*,” (IDI, Pregnant Woman Kitgum District).

#### Alcohol causes poverty and domestic violence

This theme emerged inductively from the data. Mothers associated alcohol consumption with poverty and other social ills such as domestic violence which they believe affects the wellbeing of the unborn baby and the entire family into which the baby is about to be born.

*“I highly support that all pregnant women, including me, should stop drinking alcohol because it has a lot of health effects not only on the mother but also on the unborn baby. Secondly, we all know the effect of alcohol financially. It causes a lot of poverty. A family that drinks is always poor and domestic problems are always prevalent. Children from such families don’t go to school*,” (IDI, Pregnant Woman, Pader District).

#### Alcohol can result into poor birth outcomes

On the other hand, some respondents exhibited specific comprehensive knowledge about dangers of drinking during pregnancy. They said alcohol could cause miscarriage, delayed development, brain damage, low birth weight and deformation of the baby in the womb.

*“I know that alcohol causes serious health problems both to the mother and the unborn baby. It should be stopped. Alcohol leads to impairment, brain damage, and low thinking and these are all serious problems. The baby may be born very thin and unhealthy,”* (IDI, Pregnant Woman Gulu District).

#### Alcohol can cause complications during pregnancy

Whereas some respondents had specific knowledge on dangers of maternal drinking during pregnancy, others had fragmentary information on possible dangers of drinking during pregnancy. They said alcohol use during pregnancy could result in complications during pregnancy. They further reported that the child would be born with ‘a disfigured shape of head’ if the mother drinks during pregnancy and the child could be ‘born abnormal’. Some mentioned that the ‘baby may not grow well’.

*“Children born of women who drink can have a crooked head. Some are born when they are drunk. They can’t cry like other children. They are born abnormal. They are not like other children,” (*IDI Pregnant Woman Pader District).

#### No knowledge on alcohol use during pregnancy

Some mothers admitted they were not aware that alcohol use during pregnancy was of any good or bad to the mother or unborn baby.

*“I don’t know. I have no information about it. I don’t know whether alcohol poses any threat to the baby or not*,” (IDI, Pregnant Mother, Gulu District).

#### Potential for FASD among children born of drinking mothers

Some key informants noted that they had observed undesirable characteristics among children born of mothers who imbibe alcohol during pregnancy. From the perspective of health workers, children born of drinking mothers were fatigued at birth, inactive and small for gestation age (SGA).

*“I once attended to a drank mother. She was always drank whenever she came for antenatal care. She used to have sackets of waragi in her pockets. On delivery her child was small; he looked tired and didn’t cry at birth,” (*KII, Kitgum District*).*

### Drinking alcohol as inevitable under certain circumstances

#### Alcohol as a source of livelihood

The Acholi region has some of the worst poverty indicators in the country. This theme emerged inductively. Some respondents reported that they resorted to making local brew as a source of income and inevitably find themselves consuming alcohol most of their lives even during pregnancy either to taste its tartness or to entertain their clients. Some revealed that they would be willing to abandon the trade given alternative sources of income that do not jeopardise their health. Some reportedly hail from families that brewed alcohol for a living and have been habituated into drinking alcohol since childhood. These same sentiments were echoed by health workers.

*“It is not easy to stop alcohol use once one has already started. Some have been drinking alcohol since childhood. Some come from families where brewing alcohol has been a source of income and all family members are engaged in brewing. In that case it is hard to avoid drinking alcohol,” (*KII, Kitgum District*).*

#### Comorbid conditions responsible for drinking

This theme emerged inductively from the data. Some pregnant women said that their drinking was precipitated by other comorbid conditions. Women with long term illnesses such as Human Immuno Deficiency Virus (HIV) reported that they were more at risk of drinking during pregnancy as compared to other women. They said that drinking enabled them to socialise and temporarily forget their predicament. Interviews with health workers confirm that indeed women in HIV care were more vulnerable to drinking alcohol during pregnancy as compared to their counterparts.

*“Although it is not okay for pregnant women to drink, some situations force them to do so. Even though as pregnant mothers we are advised not to drink alcohol, it is very hard for some of us to comply with the situation. Thinking of swallowing Anti-Retroviral Therapy Drugs (ARVs) daily for the rest of your life, its better you drink and forget about your HIV status and about the kind of life the children you are producing are going to have when both parents are not alive. It also helps me mix with people,” (*IDI, Pregnant Woman, Kitgum district).

#### Bewitched to drink alcohol

Some respondents reported that they did not plan to drink but still found themselves drinking. Some believed they were bewitched or cursed to drink and become a laughing stock in society.

*“Some say they have been bewitched to take alcohol. They say people who don’t wish them well such as neighbours, relatives or even friends could have cast a spell on them to drink endlessly and become a disgrace to society”* (KII, Pader District).

#### Craving alcohol

This theme emerged inductively from the data. Some of the study participants intimated that they often craved various types of alcoholic beverages during pregnancy. Astonishingly, some reportedly drank only during pregnancy because of this craving and stopped soon after delivery.

*“People have their own reasons for drinking. I see from myself most times when I am pregnant, I feel like drinking alcohol most of the time. But alcohol is a problem here. Women drink a lot of waragi and it’s a problem. Sometimes we drink because our hearts feel that we should drink, the body demands for it and me, especially, I stay alone without a husband so nobody can stop me from drinking.” (Pregnant Woman, Gulu District).*

#### Drinking during social gatherings

Other researchers have recorded drinking as part and parcel of culture [29] and we explored this theme. Cultural festivals are deeply ingrained in the Acholi culture. These include funeral rites and marriage ceremonies that take place for half of the year. Other gatherings where drinking is the norm are clan meetings and market days which are more regular. Respondents reported that during such occasions, drinking alcohol is part of these festivities. Without food and alcohol many of these festivals would be incomplete. Pregnant women are lured into drinking alcohol during such festivities. Women mentioned that family and friends expected them to fully participate in all the ceremonial activities including drinking irrespective of their pregnancy status.

*“You know attending these cultural festivals is a must. Only children are left home. All daughters-in-law must come and attend whether they are pregnant or not. They do have funeral rites after every festive season. And they prepare alcohol and food. These festivities are usually funeral rites and marriage ceremonies,” (*KII, Gulu District).

“*Access is not restricted. They have to fit in. They have to do what others are doing or be seen to be uncooperative. If you are married in a home you have to do what is expected of you in your matrimonial home. Whatever your in-laws are eating or drinking you must also participate. When there are festivals women have to participate in all the activities,” (*KII,Kitgum District).

#### Drinking pattern

Whereas some pregnant women drank singly at home, others reportedly drank in groups in bars with friends, family members or spouses as a means of socialisation. To some, these alcoholic beverages are consumed as a repast. They claim some of these drinks are nutritious especially *kwete* and when they make it they sometimes don’t prepare any other meal and the dregs are consumed by children.

### Community views about drinking during pregnancy

#### Drinking is disgraceful

In anticipation that drinking mothers would be stigmatised [8], we looked for this theme. Uncharitable, pejorative language was used by many respondents to refer to women who consume alcohol more so during pregnancy. Words such as ‘despised’, ‘disrespected’,‘irresponsible’ ‘unserious’, careless, ‘useless’, ‘disgraceful’ and ‘uncultured’ were used to describe these women. In some communities maternal drinking during pregnancy was frowned upon and these women were given name tags. This forced them to drink in hiding further compounding the problem since they couldn’t access counselling.

*“A woman who drinks is considered careless. They are actually considered even maybe useless. Imagine mature women with children drinking. It is disgraceful. She is a shame to her family. A shame to the community and a shame to herself,* “(KII, Kitgum District).

#### Alcohol is a form of socialisation

This theme emerged inductively from the data. In other communities’ especially urban settings, maternal drinking during pregnancy was tolerated especially if it was not excessive and the women were accompanied by spouses, friends or family members. This was more so in urban settings. It was regarded as an opportunity to socialise with other persons and make meaningful friendships.

#### Drinking mothers are emotionally challenged

Some respondents sympathised with drinking mothers and regarded them as persons with emotional challenges that were thwarting their lives and that of their unborn babies. These were referred for counselling. This is illustrated by the quotation below.

*“They give them advice to stop drinking, especially when pregnant. They look at it badly. You know when a woman takes alcohol it is not good. They call her and sit her down not to continue alcohol use*,” (KII, Gulu District).

#### Let drinking mothers be

This theme emerged inductively from the data. Some had a laissez faire attitude towards alcohol use during pregnancy. They felt women should be left to do what they like. Since most alcohol consumed is locally brewed and has no warning labels, they reasoned that drinking mothers should be left to drink as they like as some brews were in fact nutritious.

*“They neglect them. They let them be. It’s your life. You live it the way you like. People don’t care about them that much. Everybody is preoccupied with their own issues. People just ignore them, they just don’t give a damn.”(*KII, Gulu District*).*

### Alcohol prevention measures in communities

#### Antenatal care education

Many health workers mentioned that as part of routine antenatal care, women are educated about dos and don’ts during pregnancy including alcohol use and its dangers. But they also mentioned that some mothers report late for their first antenatal visit thereby affecting the timing of receiving the information. When asked why they reported late for antenatal care, the women said that they have to wait on their spouses for permission.

*“We teach women here at the health facility about dangers of drinking during pregnancy, although some report for ANC late*. *These women start attending antenatal care when they are like four months pregnant so they are still drinking and miss out on education sessions about alcohol use when pregnant.*”(KII, Kitgum District)

#### Local council by-Laws

In some of the study areas, the local authorities had come up with measures that sought to regulate drinking hours and prohibit consumption of certain types of alcohol that they considered dangerous. This was more so in Gulu district. According to respondents, bars are only permitted to open from 04.00p.m to midnight. Individuals found drinking during working hours were subjected to disciplinary action which included police arrests and paying fines among others. The sale, marketing and consumption of sachet alcohol was especially banned in some communities.

#### Clan meeting rebukes

Clan meetings are an intrinsic part of the Acholi culture. Every clan meets regularly and discusses several issues pertaining to the wellbeing of their people. In these meetings the people who are considered excessive drinkers are rebuked.

*“Here, there are clan meetings every month. Every clan has a meeting. If there is an emergency they call a meeting immediately. You are asked to pay a fine when you do something wrong. They meet monthly and correct whatever is going wrong in the clan e.g land disputes. Drinking alcohol also falls in there … .” (*KII, Gulu District).

#### Community dialoguing

Community dialogue meetings commonly known as BARAZAs are held in which various health issues are discussed. Community members freely share their feedback about services provided by health workers. They also have question and answer sessions about various issues in the community. Alcohol use and its dangers was mentioned as one of the issues discussed during the dialogues that are mostly funded by development partners.

#### Group and individual Counselling

Community Health Workers have also been involved in providing both individual and group counselling to women who have been identified as problem drinkers. Some of these have suffered other consequences as a result of drinking such as gender based violence. Community health workers follow up women in their communities regularly and keep speaking to them to avoid alcohol.

#### Additional challenges faced by women

##### Lack of male spousal support

This theme emerged inductively from the data. Pregnant women reported several other issues that affect them during pregnancy and predispose them to drinking. These include lack of male spousal support. Some had husbands who drink and expect them to join in even when pregnant. This is elucidated in the following extract:

*“Because of problems we may have with husbands, we decide to drink to relax a bit and forget those problems. Some husbands are drunkards and want us to drink with them. Some don’t provide for us. I am pregnant but he hasn’t bought me any maternity dress and he has money to drink. You are a woman, so I think you know men. Sometimes, we harvest our produce and the man grabs the money from us,” (IDI, Kitgum district).*

##### Maltreatment by health workers

This theme emerged inductively from the data. Some respondents reported that some health workers at health facilities were unkind to them. They said they were harshly treated by medical professionals whenever they did not meet their expectations such as reporting for Antenatal care clinics in maternity wear as they always advised them to. The women noted that some of these expectations were beyond their reach financially. The mothers revealed that they were forced to keep drinking to cope with stress.

*“Pregnant mothers don’t have food to eat, and there is no money to buy needs as told by health workers. For instance, I don’t have maternity wear and yet it's needed during ANC. Without it, the health workers quarrel with us, which makes us feel bad. We work very hard to get money. Given our condition, getting firewood is a problem for us here and you can’t eat what you feel like eating. I have to drink to forget the rude remarks from health workers,” (*IDI, Pregnant woman, Kitgum District).

##### Abusive relationships

In relation to the previous theme, this theme also emerged inductively from the data. Some pregnant mothers reported that they are trapped in abusive relationships. They said they are abused by their spouses emotionally, verbally and sometimes physically. Drinking alcohol was a temporary remedy to this challenge.

*“For me I drink because my husband is a drunkard and I feel bad when I haven’t drank and he is drank. He uses bad language to insult me so drinking helps me not to notice those insults of his. When he drinks and I don’t, I take in every insult and it hurts so much*,” (IDI, Kitgum).

##### Women’s views about anti-alcohol Services in the Community

Given that some studies before have noted prevailing strategies to avert maternal alcohol use during pregnancy [30], we explored this theme deductively. Some women did not recall receiving any information about drinking during pregnancy from any source. Some said they had received education on the dangers of drinking during pregnancy but lacked alternative remedies to treat pregnancy-related conditions for which they consumed alcohol. Others revealed that they were willing to abandon alcohol brewing and drinking given a supportive environment. Some mothers suggested the need to use mass media to continuously educate women on the dangers of maternal drinking during pregnancy. Respondents also recommended a complete ban of alcohol in the communities to minimise temptations for its use by pregnant mothers. Finally, there was a call for community role models to influence mothers by example.

## Discussion

According to the study findings, alcohol is a sociological and economically constructed reality in the Acholi sub-region. Alcohol continues to be an integral part of several ceremonies in the study area such as marriages, funerals, naming children, resolving legal disputes and market days’ festivities. It is a part of the local value system and women being part of the community find themselves consuming alcohol.

This study also reveals that women believed that alcohol use during pregnancy was advantageous. These results are in concordance with findings from a study conducted among healthcare professionals providing antenatal care in Australia some of whom reportedly recommended alcohol use to pregnant mothers to relieve stress [31]. These results are also in agreement with other previous findings that reported alcohol use during pregnancy as advantageous. In a study conducted on maternal drinking during pregnancy in western Uganda, women reported that drinking waragi, an alcoholic drink, was believed to ‘relieve heartburn’ and ‘make baby lighter’ with regard to their preference for vaginal delivery; others reportedly ‘drank beer to make the baby big’ [32]. A Ghanaian study surveying pregnant women revealed that drinking during pregnancy ‘reduces stress and ‘cleaned’ the baby in the womb or acted as an ‘appetizer’ [26]. In the United Kingdom, a study revealed public belief that light drinking in pregnancy could enhance a child’s intelligence and behaviour [33]. These beliefs may be as a result of inconclusive debate as to whether light or moderate drinking may convey harm to the unborn child’s health [34].

Whereas some women in this study believed alcohol use during pregnancy was inconsequential, others believed that it is medicinal and also cleanses the baby in the womb. Potential explanations for these beliefs include the fact that this information was passed on to the mothers from the previous generations but it also represents gaps in health promotion and chronic disease prevention.

In this study, women, especially multiparous women, said drinking during pregnancy was harmless. This may be because they haven’t experienced the perils of drinking during pregnancy. This study confirms previous findings from a qualitative study conducted among pregnant women in the United Kingdom who reported that their current drinking was influenced by experiences of previous pregnancies [35].

Women reported varied levels of knowledge on the dangers of drinking alcohol during pregnancy. In general, women were unaware or had limited knowledge of the impact of drinking on maternal and infant outcomes, such as fetal alcohol syndrome. Similarly, studies conducted in other sub-Saharan countries such as Ghana and Eastern Nigeria reveal several myths and misconceptions about the dangers of maternal drinking during pregnancy [26, 27].

In this study women reported that they found themselves in tempting situations that lured them to consume alcohol. These included participation in economic and social activities that predisposed them to drinking during pregnancy. This is similar to another study in a tribal dominated district in India where pregnant women reportedly could not restrain themselves from drinking alcohol during pregnancy because it was deeply ingrained in their culture [29].

The comorbidity between alcohol consumption during pregnancy and HIV positive status reported in this study shows that these mothers experience a significant burden of disease and other social problems. This supports previous research that linked HIV to substance abuse and poor healthcare seeking behaviours [36, 37]. Thus combined interventions addressing alcohol use during pregnancy and HIV/AIDS and a range of other social challenges that may lead to alcohol use are most appropriate.

It is also startling that unkind words were used to describe women who consume alcohol during pregnancy yet these women were expected to fully participate in cultural festivities such as clan meetings, funeral rites, marriage ceremonies and market/auction days where drinking alcohol is the norm. This supports the view that female drinking is not a common or generally accepted part of African culture due to religion and or their gender and reproductive roles [29]. Results of a recent study on alcohol and substance abuse in northern Uganda reported that women who were using alcohol and drugs were judged harshly [8]. These findings are in agreement with our results. This is not surprising since women are viewed as an embodiment of society’s moral values in many African societies and are often judged more harshly than men in the event that they indulge in any deviant behaviour.

### Study strength and limitations

The strengths of this study include use of qualitative approaches to obtain in-depth understanding of phenomena of alcohol use in this community particularly among pregnant women. The in-depth interviews provided rich descriptive data about women’s attitudes, knowledge and experiences regarding alcohol use during pregnancy. This was triangulated with information from service providers. This study discusses a topical subject and contributes to the debate about efforts aimed at reducing alcohol use among women and alcohol-exposed pregnancies such as alcohol screening and brief intervention and choices, more so within primary healthcare systems. However, this study was done in a post-conflict setting and findings may not be generalizable to other populations. Also, the majority of health workers interviewed were midwives and nurses because this cadre of staff were available at the health facilities during the study visit. It is possible that the views of some specific cadres including obstetricians, gynaecologists and anaesthetists who provide birthing and pregnancy care in this study were not adequately represented.

## Conclusions and recommendations

Pregnant mothers in post-conflict northern Uganda regard alcohol as a remedy to some of their social, economic and health challenges. It is weaved into the Acholi societal customs, and women continue drinking even during pregnancy because of the existing socio-cultural norms that promote it. The limited knowledge about the dangers of alcohol and favourable attitudes towards maternal drinking could be responsible for this practice.

Policy makers at various levels should ensure that mothers are given honest and accurate information on drinking during pregnancy so that they can make informed choices.

Both community leaders and immediate family members should be educated on the dangers of maternal drinking so that they can provide a supportive environment for women to abstain from alcohol or drink less during pregnancy while still respecting Acholi culture.

Health care providers should also screen ANC mothers for depression as it may be a proxy measure to identify women at risk of using alcohol and if need be provide individual counselling since women drink for various social challenges some of which are pregnancy-related.

Women with high levels of alcohol use should be encouraged to cut down on their alcohol use during pregnancy as much as possible, more so if they cannot or are unwilling to abstain.

Given the strong beliefs voiced by women in this study and socio-cultural factors around alcohol use, community strategies should be employed that minimize risk associated with alcohol use especially since low level alcohol use seems to be inconsistently or only weakly associated with negative maternal and infant/child outcomes.

Many healthcare providers reported that all women visiting health facilities for antenatal care are educated on dangers of maternal drinking yet these women had varying knowledge levels and conflicting views regarding maternal alcohol use during pregnancy. Future studies should investigate in detail the content and consistency of messages shared by health workers about pregnancy and drinking.

## Data Availability

All relevant data are within the paper. If further data is needed, it could be accessed from the first author upon request via email at agiresaasi@gmail.com.

## Appendix 1

**Table 1:** Showing Sample Questions in Data Collection Guides

Questions in Health Workers’ KII Guide	Questions in Pregnant Women’s In-depth Interview Guide
Which are some of the issues affecting pregnant mothers in your community?	Which are some of the common challenges that affect women in this community?
Would you say they have a problem of alcohol use?	Do you take alcohol?
How do people perceive alcohol use for women in this community? (Probe for pregnant women)	When did you start consuming alcohol?
What pattern have you observed among women who use alcohol in this community?	With who do you take it? Where?
Is alcohol use common among pregnant women in your community?	What kind of alcohol do you usually consume?
What are the most common types of alcohol consumed in this community?	Why do you think some women like yourself drink during pregnancy (Excuses and reasons)?
According to you, are there particular behaviours / life styles/ risk factors that you observe in this community which are associated to alcohol use during pregnancy? (Probe for age, education level, parity, socio economic status, related behaviour etc.)	Do you think it is okay for pregnant women like yourself to consume alcohol? (why or why not probe further)
Can you tell me some of the telling symptoms/ characteristics of pregnant women who use alcohol in your community during pregnancy?	Do you see alcohol posing a big threat to women who drink during pregnancy like yourself? (Probe on the unborn babies, on pregnant mothers).
Why do these women consume alcohol? (reasons and excuses)	Do you think there is any danger in consuming alcohol during pregnancy?
Are there any precautionary measures people can take in the context of this community to prevent such alcohol use during pregnancy? [How can women here avoid alcohol use during pregnancy? (Probe for measures in place at health facilities and in communities).	What dangers does it cause if any?
Why do you think pregnant women in this community do not take these measures to prevent alcohol use?	If you consider it serious, are there any precautionary measures you are taking to stop drinking?
Do you see alcohol consumption as posing a big threat for women in this community? (Probe for related ailments/burdens).	Has somebody ever talked to you about stopping drinking during pregnancy? (Probe for relatives, health worker, spouse religious leader etc.)
Are you aware of any laws regulating alcohol? If yes, do you consider them effective? Can something better be done to that situation either by the government, municipal council or the community/health systems?	Do you think alcohol should be discouraged for all women? (probe for all women, pregnant women, women of child bearing age)
Are there any health related issues with regard to alcohol use and pregnancy that we have not discussed but which you would have wanted us to discuss?	
	Why do you think women in this community like your self do not take these measures to prevent alcohol use during pregnancy?
	What should be done to prevent alcohol use during pregnancy and its consequences (probe – the community, health workers and other stakeholders)
	Are you aware of any measures or laws regulating alcohol use during pregnancy? If yes, do you consider them effective? Can something better be done to that situation either by the government, municipal council or the community, health systems?

Questionnaires explored pregnant women’s attitudes and experiences related to alcohol use during pregnancy, they also assessed health worker views on alcohol use during pregnancy, drinking patterns and prevailing interventions in the community

## Appendix 2

**Table 2:** Showing Demographic Characteristics of Women who participated in the Study

Variable	Frequency(N)	Per cent (%)
**Age group**		
15–19	5	16.6
20–25	3	10
26–30	14	46
31–35	7	23
36–40	1	3.3
**Occupational Status**		
Formal employment	2	6.6
Trader	3	10
Alcohol Brewer	3	10
Farmer	19	63
No employment	3	10
**Marital Status**		
Cohabiting	18	60
Married	8	26.6
Divorced/Separated	4	13.3
**Education Level**		
Never attended school	2	6.6
Primary	17	56.6
Secondary	9	30
Tertiary	2	6.6
**Income**		
No Income	9	30
5000–50,000	11	36.6
60,000–100,000	3	10
110,000 -300,000	5	16.6
310,000–500,000	1	3.3
More than 1 million	1	3.3

Almost half of the respondents belonged to the 26–30 age group. About two thirds are farmers. Slightly more than half have attained primary level education. Most of the respondent earned between 110,000 to 300,000Shs monthly

## Appendix 3

**Table 3:** Table showing Summary of Study Participants and Methods of Data Collection

Participant Category	Method of Data Collection	Study District			Total
		Gulu	Kitgum	Pader	
		Male Female	Male Female	Male Female	
Health workers	Key Informant Interviews	02 07	04 07	02 08	30
Pregnant women	In-depth Interviews	00 10	00 10	00 10	30

Over 70% of health workers interviewed were female. Thirty pregnant women who reported alcohol use during pregnancy were interviewed

## Abbreviations

ANC: Antenatal Care
ARVs: Anti-retroviral Therapy
FASD: Fetal Alcohol Spectrum Disorders
IDI: In-depth Interview
IDP: Internally Displaced Peoples
KII: Key Informant Interviews
OPD: Outpatient Department
TFA: Thematic Framework Analysis
UNCST: Uganda National Council for Science and Technology
VHTs: Village Health Teams
